# Disparities in rheumatoid arthritis disease activity according to gross domestic product in 25 countries in the QUEST–RA database

**DOI:** 10.1136/ard.2009.109983

**Published:** 2009-07-31

**Authors:** T Sokka, H Kautiainen, T Pincus, S Toloza, G da Rocha Castelar Pinheiro, J Lazovskis, M L Hetland, T Peets, K Immonen, J F Maillefert, A A Drosos, R Alten, C Pohl, B Rojkovich, B Bresnihan, P Minnock, M Cazzato, S Bombardieri, S Rexhepi, M Rexhepi, D Andersone, S Stropuviene, M Huisman, S Sierakowski, D Karateev, V Skakic, A Naranjo, E Baecklund, D Henrohn, F Gogus, H Badsha, A Mofti, P Taylor, C McClinton, Y Yazici

**Affiliations:** 1 Jyväskylä Central Hospital, Jyväskylä; Medcare Oy, Äänekoski, Finland; 2 Unit of Family Practice, Jyväskylä Central Hospital, Jyväskylä, and ORTON, Rehabilitation Unit, Helsinki, Finland; 3 NYU Hospital for Joint Diseases, New York, NY, USA; 4 Hospital San Juan Bautista, Catamarca, Argentina; 5 Universidade do Estado do Rio de Janeiro, Rio de Janeiro, Brazil; 6 Riverside Professional Center, Sydney, NS, Canada; 7 Copenhagen Univ Hospital at Hvidovre, Hvidovre, Denmark; 8 East-Tallinn Central Hospital, Tallinn, Estonia; 9 North Karelia Central Hospital, Joensuu, Finland; 10 Dijon University Hospital, University of Burgundy and INSERM U887, Dijon, France; 11 University of Ioannina, Ioannina, Greece; 12 Schlosspark-Klinik, Berlin, Germany; 13 Polyclinic of the Hospitaller Brothers of St. John of God in Budapest, Budapest, Hungary; 14 St. Vincent University Hospital, Dublin, Ireland; 15 Our Lady's Hospice, Dublin, Ireland; 16 Santa Chiara Hospital, Pisa, Italy; 17 Rheumatology Department, Pristine, Kosovo; 18 Pauls Stradina Clinical University Hospital, Riga, Latvia; 19 Institute of Experimental and Clinical Medicine at Vilnius University, Vilnius, Lithuania; 20 Sint Franciscus Gasthuis Hospital, Rotterdam, Netherlands; 21 Medical University in Bialystok, Bialystok, Poland; 22 Early Arthritis Department, Institute of Rheumatology of Russian Academy of Medical Sciences, Moscow, Russia; 23 Rheumatology Institut, Niska Banja, Serbia; 24 Hospital de Gran Canaria Dr. Negrin, Las Palmas, Spain; 25 Uppsala University Hospital, Uppsala, Sweden; 26 Gazi University Medical Faculty, Ankara, Turkey; 27 Dubai Bone and Joint Center, Dubai, United Arab Emirates; 28 American Hospital Dubai, Dubai, United Arab Emirates; 29 University Medical Faculty, Charing Cross Hospital, London, UK

## Abstract

**Objective:**

To analyse associations between the clinical status of patients with rheumatoid arthritis (RA) and the gross domestic product (GDP) of their resident country.

**Methods:**

The Quantitative Standard Monitoring of Patients with Rheumatoid Arthritis (QUEST–RA) cohort includes clinical and questionnaire data from 6004 patients who were seen in usual care at 70 rheumatology clinics in 25 countries as of April 2008, including 18 European countries. Demographic variables, clinical characteristics, RA disease activity measures, including the disease activity score in 28 joints (DAS28), and treatment-related variables were analysed according to GDP per capita, including 14 “high GDP” countries with GDP per capita greater than US$24 000 and 11 “low GDP” countries with GDP per capita less than US$11 000.

**Results:**

Disease activity DAS28 ranged between 3.1 and 6.0 among the 25 countries and was significantly associated with GDP (r  =  −0.78, 95% CI −0.56 to −0.90, r^2^  =  61%). Disease activity levels differed substantially between “high GDP” and “low GDP” countries at much greater levels than according to whether patients were currently taking or not taking methotrexate, prednisone and/or biological agents.

**Conclusions:**

The clinical status of patients with RA was correlated significantly with GDP among 25 mostly European countries according to all disease measures, associated only modestly with the current use of antirheumatic medications. The burden of arthritis appears substantially greater in “low GDP” than in “high GDP” countries. These findings may alert healthcare professionals and designers of health policy towards improving the clinical status of patients with RA in all countries.

Health disparities, including high mortality rates, are associated with low socioeconomic status in many specific diseases in many countries.^1^ ^2^ ^3^ ^4^ ^5^ ^6^ Furthermore, differences in gross domestic product (GDP) in different countries are associated significantly with differences in mortality rates among countries.^7^ ^8^ ^9^ Most reports of these observations are based on surveys and national databases, with relatively limited information from clinical settings based on physical examination, laboratory tests, medications and patient self-report information concerning functional status, pain, psychosocial distress, etc, to understand further the basis for these disparities. Furthermore, little is known concerning associations of GDP and clinical outcomes of chronic disabling musculoskeletal conditions such as rheumatoid arthritis (RA).

A multinational database Quantitative Standard Monitoring of Patients with Rheumatoid Arthritis (QUEST–RA)^10^ ^11^ was established to assess 100 unselected consecutive patients with RA per clinic and included 25 countries by April 2008. Considerable variation was observed in clinical status in different countries according to most clinical measures, whether derived from the physician, patient or laboratory, as well as the composite RA disease activity score in 28 joints (DAS28) index.^12^ In this report, we compare demographic characteristics, RA disease activity measures and treatment-related variables between “high GDP” and “low GDP” countries, and analyse associations between DAS28 and GDP.

## Patients and methods

### Establishment of database

QUEST–RA was established in 2005 with the objectives of promoting quantitative assessment in usual rheumatology care, and to develop a baseline cross-sectional database of consecutive RA patients seen outside of clinical trials in regular care in many countries. Three or more rheumatologists were asked to enroll 100 consecutive unselected patients in each country.^10^ As of April 2008, the programme enrolled 6004 patients from 71 sites in 25 countries, including Argentina, Brazil, Canada, Denmark, Estonia, Finland, France, Germany, Greece, Hungary, Ireland, Italy, Kosovo, Latvia, Lithuania, The Netherlands, Poland, Russia, Serbia, Spain, Sweden, Turkey, United Arab Emirates, the UK and the USA.^11^

The study was carried out in compliance with the Helsinki Declaration. Ethics Committees or Internal Review Boards of participating institutes approved the study, and a written informed consent was obtained from the patients.

### Physician assessment measures

All patients were assessed according to a standard protocol to evaluate RA.^13^ The physician review included a formal examination to count the number of swollen and tender joints, report of radiographic erosions, erythrocyte sedimentation rate (ESR) and rheumatoid factor (RF) positivity according to local reference values. Physicians reported the use of disease-modifying antirheumatic drugs (DMARD), including: (1) the interval between the first symptoms and the start of the first DMARD; (2) the number of DMARD ever used; (3) the use of methotrexate, oral glucocorticoids, biological agents and other DMARD and (4) the start and stop dates for each DMARD used. The total duration of DMARD therapy as a percentage of disease duration was computed.

### Patient self-report questionnaire measures

Patients completed a 4-page self-report questionnaire, which included demographic data, physical function, pain, global estimate and psychological distress. Demographic data included date of birth, gender, years of formal education, race and smoking status, as well as weight and height, to calculate body mass index (BMI). A standard health assessment questionnaire (HAQ)^14^ assesses physical function in activities of daily living with four response categories: 0, without any difficulty; 1, with some difficulty; 2, with much difficulty; 3, unable to do. Visual analogue scales (0  =  best to 10  =  worst) were completed for pain and patient estimate of his/her global health (GH). Psychological distress was assessed as the capacity to cope with stress, anxiety and depression, queried and calculated in the HAQ format for psychological HAQ scored 0–3.^15^

### Disease activity score

The DAS28^12^ is the most widely used overall measure for RA disease activity, comprising two physical examination measures, a swollen 28-joint count (SJC28) and a tender 28-joint count (TJC28); one laboratory measure, ESR; and one patient self-report measure, a global estimate of health status (GH). DAS28 ranges from 0 to 9.1 (low–high), calculated from the formula 0.56*sqrt(TJC28) + 0.28*sqrt(SJC28) + 0.70*Ln(ESR) + 0.014*(GH).

### Gross domestic product

The GDP of each country in 2005 was obtained from a database of the International Monetary Fund^16^ and is expressed as UD$1000 or Euros (€) per capita (with an exchange rate of US$1.25  =  €1.0 in 2005). GDP ranged from 3.5 to 49, including 14 “high GDP” countries with GDP per capita greater than US$24 000 and 11 “low GDP” countries with GDP per capita less than US$11 000.

### Statistical methods

Descriptive results for each country are presented as mean, median and percentages. Comparisons of demographic variables, clinical characteristics, RA disease activity measures and treatment-related variables within and/or between “high GDP” and “low GDP” countries were performed using parametric and non-parametric tests for continuous variables and the χ^2^ test for categorical variables. Linear regression models were applied to analyse possible associations between DAS28 and GDP.

## Results

### Patients

The 6004 patients in the QUEST–RA database, from 71 clinics in 25 countries, represent a typical RA cohort in demographic features, with a mean age of 56 years, 79% women, and a mean disease duration of 11 years (table 1). Patients were younger and more likely to be women in “low GDP” compared with “high GDP” countries, whereas years of formal education, BMI and disease duration were similar (table 1). Patient characteristics are presented for each European and non-European country, in descending order of GDP in table 2.

**Table 1:** Comparison of patient characteristics, RA disease activity measures and treatment-related variables in the QUEST–RA study in “high GDP” and “low GDP” countries

	Total	“High GDP” countries	“Low GDP” countries	p Value*
N	6004	3719	2285	
Patient characteristics				
Female, %	79%	75%	86%	<0.001
Age, years, mean	56	58	54	<0.001
Education, years, median	11	11	11	0.16
Smoking now, %	17%	19%	14%	<0.001
BMI, mean	26	26	26	0.63
Disease duration, years, mean	11	11	11	0.038
Disease activity measures				
DAS28 (0–9.1), mean	4.2	3.7	5.1	<0.001
Physician				
SJC (0–28), median	2	1	4	<0.001
TJC (0–28), median	4	2	8	<0.001
Physician global (0–10), median	2.4	1.7	3.7	<0.001
Patient self-report				
HAQ physical function (0–3) median	0.88	0.75	1.3	<0.001
Pain VAS (0–10), median	4.1	3.3	4.9	<0.001
Patient global VAS (0–10), median	4.2	3.2	4.9	<0.001
Psychological HAQ (0–3), mean	0.82	0.7	1.3	<0.001
Laboratory/imaging				
ESR, median	22	18	30	<0.001
RF-positive, %	74%	72%	76%	0.001
Erosive disease, %	63%	58%	72%	<0.001
In patients with disease duration ⩽2 years		23%	40%	<0.001
In patients with disease duration ⩾10 years		79%	84%	0.001
Treatment-related variables				
Patients taking DMARD ever, %	97%	97%	97%	0.75
Delay to start a DMARD, months, median	9	9	9	0.063
No of DMARD ever taken, mean	2.7	2.8	2.5	<0.001
Methotrexate ever, % of patients	85%	86%	84%	<0.001
Methotrexate now, % of patients	63%	63%	62%	0.36
Prednisone ever, % of patients	71%	66%	80%	<0.001
Prednisone now, % of patients	49%	42%	60%	<0.001
Biologicals ever, % of patients	23%	31%	9.4%	<0.001
Biologicals now, % of patients	18%	25%	7.5%	<0.001
Any DMARD, % of disease duration	72%	75%	67%	0.085
Methotrexate, % of disease duration	31%	32%	30%	0.18
Biologicals, % of disease duration	3.9%	5.6%	1.8%	0.001

*Unadjusted p values are presented. BMI, body mass index; DAS28, disease activity score in 28 joints; DMARD, disease-modifying antirheumatic drug; ESR, erythrocyte sedimentation rate; GDP, gross domestic product; HAQ, health assessment questionnaire; RA, rheumatoid arthritis; RF, rheumatoid factor; SJC, swollen joint count; TJC, tender joint count; VAS, visual analogue scale.

**Table 2:** Patient characteristics in the QUEST–RA study in Europe and other countries, in descending order of GDP

Country	GDP 2005 per capita (US$1000)	Sites	Patients	Women, %	Age, years, mean	Education, years, median	Smoking now, %	BMI, mean	Disease duration, years, mean
Europe									
Ireland	48.7	3	240	64	56	12	24	25	11
Denmark	47.9	3	301	77	58	10	31	25	12
Sweden	39.6	3	260	72	59	10	19	25	12
The Netherlands	38.9	3	317	66	59	11	22	26	9
Finland	37.3	3	304	72	59	9	15	27	14
UK	37.3	3	145	78	60	12	19	25	15
France	35.0	4	389	78	55	10	19	25	13
Germany	33.9	2	225	84	59	10	13	26	13
Italy	30.5	4	336	78	61	8	16	25	10
Spain	26.1	3	302	74	60	10	17	26	11
Greece	25.6	3	300	76	58	12	15	26	12
Hungary	10.9	3	153	87	58	12	24	25	13
Estonia	10.3	3	168	85	56	12	14	26	12
Poland	8.0	7	642	87	53	12	11	25	12
Lithuania	7.5	2	300	83	54	13	7	26	11
Latvia	7.0	1	79	80	53	13	18	27	13
Serbia	3.5	1	100	88	59	8	18	26	10
Kosovo	3.5	1	100	84	55	8	5	28	8
Non-Europe									
USA	41.9	3	301	73	57	13	20	28	9
Canada	35.2	1	100	79	58	12	30	27	12
UAE	32.4	2	199	86	45	15	6	26	6
Russia	5.3	3	73	85	54	13	15	26	6
Turkey	5.0	3	309	86	52	5	13	27	12
Brazil	4.8	5	115	89	52	8	13	25	8
Argentina	4.7	2	246	90	51	9	21	26	10
Total		71	6004	79	56	11	17	26	11

BMI, body mass index; GDP, gross domestic product; UAE, United Arab Emirates.

### Disease activity

Patients in “low GDP” countries had statistically significantly higher disease activity levels in all individual disease activity measures, including physician-derived measures, patient self-report scores and laboratory measures, as well as the composite index DAS28 (table 1). The percentage of patients with positive RF varied between 61% and 90% between countries (table 3), although the overall difference was only 4% between “high GDP” and “low GDP” countries (table 1). Erosive disease was less prevalent in “high GDP” countries, with differences of 14%, although with differences of only 5% in patients with more than 10 years of disease (table 1).

**Table 3:** RA measures in the QUEST–RA study in Europe and other countries, in descending order of GDP

Country	GDP 2005 per capita (US$1000)	DAS28 (0–9.1), mean	SJC (0–28), median	TJC (0–28), median	Physician global (0–10), median	HAQ physical function (0–3), median	Pain VAS (0–10), median	Patient global VAS (0–10), median	PS-HAQ (0–3), mean	ESR, median	RF-positive, %	Erosive disease, %
Europe												
Ireland	48.7	4.1	3	4	1.6	0.75	3.4	2.9	0.61	18	80	60
Denmark	47.9	3.4	1	2	1.3	0.63	2.6	2.8	0.53	14	73	64
Sweden	39.6	3.8	2	2	1.4	0.88	3.3	3.3	0.56	19	82	60
The Netherlands	38.9	3.1	1	0	0.7	0.75	2.5	2.7	0.54	15	69	44
Finland	37.3	3.3	1	1	0.9	0.63	2.8	2.8	0.46	13	75	56
UK	37.3	4.0	1	4	1.8	0.88	4.1	3.6	0.69	19	81	69
France	35.0	3.7	1	2	2.5	0.88	3.9	3.6	0.90	16	75	76
Germany	33.9	4.4	3	4	3.0	0.75	5.0	4.9	0.81	20	61	78
Italy	30.5	4.5	2	4	3.0	1.00	4.9	5.0	0.97	28	71	38
Spain	26.1	3.5	1	1	1.2	0.88	3.1	3.6	0.88	17	73	60
Greece	25.6	3.4	0	1	1.1	0.25	2.3	2.0	0.77	23	52	71
Hungary	10.9	5.1	5	7	3.3	1.38	5.2	5.1	1.08	26	93	93
Estonia	10.3	4.7	4	6	3.0	1.13	4.3	4.8	0.95	24	68	86
Poland	8.0	5.3	6	9	4.3	1.38	5.0	4.8	0.97	31	70	62
Lithuania	7.5	5.5	3	12	4.1	1.38	5.2	5.3	1.01	29	78	79
Latvia	7.0	5.2	4	8	4.9	1.38	5.1	5.7	0.93	26	85	97
Serbia	3.5	5.9	6	18	4.6	1.63	5.1	5.3	1.38	28	71	80
Kosovo	3.5	6.0	6	16	4.7	1.63	5.1	5.0	1.47	45	81	99
Non-Europe												
USA	41.9	3.3	2	1	1.9	0.63	3.2	2.6	0.47	14	71	36
Canada	35.2	4.1	2	4	1.8	1.00	4.6	4.0	0.69	21	83	45
UAE	32.4	4.3	3	3	2.6	0.63	3.7	2.8	0.69	24	75	47
Russia	5.3	5.0	5	6	3.4	1.13	3.5	4.5	1.21	24	75	60
Turkey	5.0	4.2	0	2.5	2.0	0.88	4.2	4.6	0.94	30	68	56
Brazil	4.8	4.2	3	2	1.5	0.63	3.2	3.5	0.71	28	80	63
Argentina	4.7	5.3	9	10.5	4.7	1.00	5.0	4.7	1.13	30	90	68
Total		4.2	2	4	2.4	0.88	4.1	4.2	0.82	22	74	63

DAS28, disease activity score in 28 joints; ESR, erythrocyte sedimentation rate; GDP, gross domestic product; HAQ, health assessment questionnaire; Physician global, physician global estimate of status; PS-HAQ, psychological HAQ; RA, rheumatoid arthritis; RF, rheumatoid factor; SJC, swollen joint count; TJC, tender joint count; UAE, United Arab Emirates; VAS, visual analogue scale.

### Drug therapies

DMARD were taken by 88–100% of all patients in the 25 countries (table 4), with no differences between “high GDP” and “low GDP” countries (table 1); the mean number of DMARD was 2.7. The median delay between first symptoms and initiation of the first DMARD ranged from less than 6 months in three countries to one year or more in 10 countries (table 4), with no significant difference between “high GDP” and “low GDP” countries (table 1). Methotrexate was taken by 69–98%, prednisone by 30–97% and biological agents by 1–54% of patients. DMARD were taken for less than 50% of disease duration in the UK, Ireland, Hungary, Latvia, Lithuania and Argentina, and for more than 100% in Finland, Greece and Brazil—percentages greater than 100 indicate the simultaneous use of two or more DMARD. Methotrexate covered 21–57% of disease duration and the use of biologic agents covered 0.2–14% (table 4).

**Table 4:** GDP and treatment-related variables in the QUEST–RA study in Europe and other countries, in descending order of GDP

Country	GDP 2005 per capita US$1000	Patients taking DMARD ever, %	Delay to start DMARD, months, median	No of DMARD ever taken	Methotrexate ever, % of patients	Prednisone ever, % of patients	Biologicals ever, % of patients	Any DMARD, % of disease duration	Methotrexate, % of disease duration	Biologicals, % of disease duration
Europe										
Ireland	48.7	98.0	10.0	2.4	93	71	42	55.7	28.9	6.6
Denmark	47.9	99.0	10.0	2.8	86	44	23	65.6	25.5	2.9
Sweden	39.6	94.0	12.0	2.8	84	69	33	73.7	31.1	7.2
The Netherlands	38.9	99.0	5.0	2.3	92	30	23	87.6	45.0	4.4
Finland	37.3	100.0	7.0	4.0	86	74	17	109.6	25.7	2.2
UK	37.3	95.0	12.0	2.4	83	54	20	51.3	24.6	1.9
France	35.0	99.0	8.0	3.7	87	83	53	77.0	31.0	8.9
Germany	33.9	92.0	15.0	2.7	80	54	29	66.5	28.8	6.3
Italy	30.5	96.0	9.0	2.4	81	72	27	66.9	28.9	2.9
Spain	26.1	98.0	13.0	2.8	87	68	27	67.4	30.9	4.4
Greece	25.6	100.0	7.0	2.9	94	94	54	104.2	50.8	14.6
Hungary	10.9	96.0	12.0	2.6	85	58	16	54.3	20.1	0.7
Estonia	10.3	98.0	12.0	2.8	83	76	1	67.4	23.8	0.3
Poland	8.0	99.0	4.0	3.0	89	80	10	64.0	26.3	0.9
Lithuania	7.5	96.0	13.0	2.3	84	97	11	45.3	21.2	1.7
Latvia	7.0	98.0	24.0	2.2	92	79	23	52.9	28.0	1.6
Serbia	3.5	96.0	11.0	2.0	69	88	2	64.6	25.2	0.3
Kosovo	3.5	100.0	3.0	2.0	82	95	1	82.4	36.8	0.2
Non-Europe										
USA	41.9	98	9.0	2.1	85	77	33	83.8	39.5	8.5
Canada	35.2	95	11.0	3.0	76	62	28	77.6	22.1	5.0
UAE	32.4	88	11.0	1.8	73	56	10	70.2	31.3	1.9
Russia	5.3	88	10.0	1.8	73	41	18	64.0	28.6	9.7
Turkey	5	99	12.0	2.4	89	75	7	75.3	33.4	0.7
Brazil	4.8	100	8.0	3.0	98	81	27	115.7	57.6	3.7
Argentina	4.7	88	13.0	1.5	68	83	3	46.3	24.5	0.5
Total		97	9	2.7	85	71	23	71.6	30.8	3.9

DMARD, disease-modifying antirheumatic drug; GDP, gross domestic product; UAE, United Arab Emirates.

Differences in the DAS28 scores of patients who were taking or not taking methotrexate were small within “high GDP” countries (3.6 vs 3.8) and “low GDP” countries (5.1 vs 5.2) but large between “high GDP” versus “low GDP” countries (p<0.001; table 5). Similar patterns were seen for patients who were currently taking or not taking prednisone. Patients who were taking or not taking biological agents in “high GDP” countries had similar disease activity levels of 3.7, whereas in “low GDP” countries those who were taking biological agents had a statistically significantly lower mean DAS28 of 4.4 compared with patients who were not taking biological agents (DAS28 5.2; p<0.001).

**Table 5:** Disease activity in “high GDP” and “low GDP” countries, according to current medications

	“High GDP” countries	“Low GDP” countries	p Value†
	DAS28, mean	DAS28, mean	
Methotrexate			
Taking methotrexate now	3.6	5.1	<0.001
No methotrexate now	3.8	5.2	<0.001
p Value*	0.003	0.028	
Prednisone			
Taking prednisone now	3.9	5.3	<0.001
No prednisone now	3.5	4.8	<0.001
p Value*	0.001	0.001	
Biologicals			
Taking biologicals now	3.7	4.4	<0.001
No biologicals now	3.7	5.2	<0.001
p Value*	0.63	<0.001	

*Comparison within “high GDP”/“low GDP” countries. †Comparison between “high GDP”/“low GDP” countries. DAS28, disease activity score in 28 joints; GDP, gross domestic product.

### Linear regression models for GDP and disease activity

**Figure 1:**
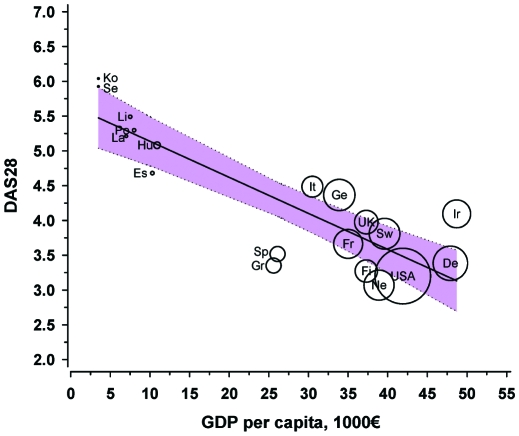
Association between gross domestic product per capita (GDP) and disease activity score in 28 joints (DAS28) in 18 European countries and the USA in the QUEST–RA study. The correlation of GDP with DAS28 is r  =  −0.85 (95% CI −0.63 to −0.94); indicated with colour. GDP is expressed as 1000€ per capita. DAS28 ranges from 0 to 9.1 (low–high disease activity). The area of the disk is proportional to the total national health expenditure of each country in 2004. De, Denmark; Es, Estonia; Fi, Finland; Fr, France; Ge, Germany; Gr, Greece; Hu, Hungary; Ir, Ireland; It, Italy; Ko, Kosovo; La, Latvia; Li, Lithuania; Ne, The Netherlands; Po, Poland; Se, Serbia; Sp, Spain; Sw, Sweden.

The mean DAS28 varied among countries from 3.1 to 6.0 (table 2), and was associated significantly with GDP (r  =  −0.78, 95% CI −0.56 to −0.90, r^2^  =  61%); a stronger association (r  =  −0.85, 95% CI −0.63 to −0.94, r^2^  =  72%) was seen when only 18 European countries were included (fig 1; data from USA presented as a reference in the figure). Among European countries, an increase of GDP per 1000€ translates to a decrease of DAS28 of −0.10 (95% CI −0.061 to −0.13).

## Discussion

These data from the QUEST–RA database indicate that the burden of RA is substantially greater in “low GDP” than in “high GDP” countries, within and outside of Europe. These results are consistent with data concerning other chronic diseases and general health in different countries.^7^ ^8^ ^9^ ^17^ The burden of chronic diseases, recognised as an important neglected global health issue,^18^ is greatest in low and middle income countries.^19^ Most chronic diseases occur at substantially higher frequency in individuals of low socioeconomic status, particularly before the age of 65 years.^1^ ^2^ ^3^ ^4^ ^5^ ^6^ Poor outcomes of RA including mortality are associated with low socioeconomic status.^5^ ^20^

Associations of low socioeconomic status and poor health, within and between countries, are regarded in most medical literature as based primarily on limited access to medical care and problems in the structure of clinical care,^21^ ^22^ ^23^ reflecting a “biomedical model”, the dominant paradigm of 20th century medicine.^23^ ^24^ However, in the present study, all patients had access to a rheumatologist, and 88–100% of patients had taken DMARD, including 69–98% who had taken methotrexate. Treatment-related variables were quite similar between “high GDP” and “low GDP” countries, including 97% of patients who had used DMARD, a delay of 9 months to the start of DMARD after first symptoms, the number of DMARD ever taken (2.8 and 2.5); 86% and 84% had ever used methotrexate and the use of methotrexate covered similarly 32% and 30% of disease duration in “high GDP” and “low GDP” countries. Disease activity levels appeared much more associated with the wealth of the country than current medications (table 5). The current use of biological agents appeared to have a greater inverse association with disease activity in “low GDP” than in “high GDP” countries (table 5).

GDP explained 61% of the variation in DAS28 scores, consistent with extensive observations of differences in health status and mortality in different nations according to a nation’s wealth.^7^ ^8^ ^9^ Differences in DAS28 scores according to GDP are at least twice as great as improvements in DAS28 achieved in any clinical trial of the most powerful biological agents. The data echo a suggestion made by Virchow in 1848 that “the improvement of medicine would eventually prolong human life, but improvement of social conditions could achieve this result now more rapidly and more successfully.”^25^

Many psychological constructs, including stress, anxiety, depression, limited social support, learned helplessness and lack of optimism, are prognostic of higher mortality rates in individuals.^23^ ^26^ ^27^ ^28^ These reports suggest that associations of low socioeconomic status with poor health and high mortality may be explained as much or ever better by psychosocial and behavioural variables as by “medical” variables, according to a “biopsychosocial model” to complement a biomedical model.^23^ ^24^ ^27^ ^28^ In the present study, psychological distress appeared significantly greater in “low GDP” than in “high GDP” countries.

Several limitations are seen in this study. First, cross-sectional QUEST–RA data cannot address definitively whether the biology of RA is more severe in less wealthy versus wealthy countries. GDP may serve as a surrogate marker for many variables, some related and others unrelated directly to medical care.

Second, a limited number of patients were included per country. Nonetheless, results were quite similar among clinics within each country.^10^ Furthermore, it is not possible to obtain optimal clinical information on large numbers of patients, with accurate physical examination and physician-recorded diagnosis for diseases such as RA, using only surveys. RA is not diagnosed according to a single “gold standard” measure as seen for hypertension or diabetes, but according to a set of measures, requiring a knowledgeable clinician.

Third, it is possible that only RA patients with poor clinical status visit clinics in countries with lower GDP, and patients with better status in rich countries seek medical care. While this possibility cannot be excluded, the study was designed to incorporate a cross section of patients seen in various countries. Clinical trials in patients with RA tend to be performed in countries with lower GDP at this time, as most patients in countries with higher GDP do not meet inclusion criteria,^29^ indicating the likelihood of an accurate representation of the present study.

These results illustrate the potential value of collaborative databases, with identical database architecture, to provide an opportunity to study associations between disease characteristics, therapies and outcomes at many sites in many countries with different economic levels. The QUEST–RA database may appear somewhat complex, although the collected data were regarded as consistent with appropriate good patient care. The only payments made to physicians were to cover the costs of materials and mailing. Similar efforts could be conducted at low cost for many diseases and add useful information regarding clinical status, prognosis and monitoring.

The status of patients with RA has been found to be significantly better in recent years than in previous decades.^30^ ^31^ ^32^ However, almost all of the published data have been derived from western European and north American nations. The data presented in this report, indicating disparities with other countries, present an important challenge. While it is possible that other nations may “catch up” over the next decade to advances in the wealthier nations at this time, disparities may continue to exist. Public health efforts would appear potentially to be as important as the introduction of new therapies to treat RA.^33^

Further research is needed to understand better the structure (demographic, macroeconomic), process (clinical and treatment variables) and outcomes (mortality, functional and work status), which may contribute to differences in quality of care for patients with RA in different countries. Recognition of major differences in outcomes can lead to more informed efforts to improve the structure and process of care. The ultimate goal of these efforts is to improve outcomes for patients with RA in all countries.
